# Transient oscillatory dynamics of interferon beta signaling in macrophages

**DOI:** 10.1186/1752-0509-7-59

**Published:** 2013-07-09

**Authors:** Inna Pertsovskaya, Elena Abad, Núria Domedel-Puig, Jordi Garcia-Ojalvo, Pablo Villoslada

**Affiliations:** 1 Center of Neuroimmunology, Institut d'Investigacions Biomèdiques August Pi i Sunyer (IDIBAPS), Hospital Clinic of Barcelona, Barcelona, Spain; 2 Department of Experimental and Health Sciences, Universitat Pompeu Fabra, Barcelona, Spain; 3 Department of Physics and Nuclear Engineering, Universitat Politecnica de Catalunya, Spain

**Keywords:** Type I interferon pathway, Interferon-beta, Ordinary differential equation, Oscillations, Multiple sclerosis, Immunotherapy

## Abstract

**Background:**

Interferon-beta (IFN-beta) activates the immune response through the type I IFN signaling pathway. IFN-beta is important in the response to pathogen infections and is used as a therapy for Multiple Sclerosis. The mechanisms of self-regulation and control of this pathway allow precise and environment-dependent response of the cells in different conditions. Here we analyzed type I IFN signaling in response to IFN-beta in the macrophage cell line RAW 264.7 by RT-PCR, ELISA and xMAP assays. The experimental results were interpreted by means of a theoretical model of the pathway.

**Results:**

Phosphorylation of the STAT1 protein (pSTAT1) and mRNA levels of the pSTAT1 inhibitor SOCS1 displayed an attenuated oscillatory behavior after IFN-beta activation. In turn, mRNA levels of the interferon regulatory factor IRF1 grew rapidly in the first 50–90 minutes after stimulation until a maximum value, and started to decrease slowly around 200–250 min. The analysis of our kinetic model identified a significant role of the negative feedback from SOCS1 in driving the observed damped oscillatory dynamics, and of the positive feedback from IRF1 in increasing STAT1 basal levels. Our study shows that the system works as a biological damped relaxation oscillator based on a phosphorylation-dephosphorylation network centered on STAT1. Moreover, a bifurcation analysis identified translocation of pSTAT1 dimers to the nucleus as a critical step for regulating the dynamics of type I IFN pathway in the first steps, which may be important in defining the response to IFN-beta therapy.

**Conclusions:**

The immunomodulatory effect of IFN-beta signaling in macrophages takes the form of transient oscillatory dynamics of the JAK-STAT pathway, whose specific relaxation properties determine the lifetime of the cellular response to the cytokine.

## Background

Type I interferons, such as interferon alfa and beta, are cytokines that represent a first-line endogenous defense mechanism in response to viruses and bacterial infections, are secreted by many cell types (e.g. lymphocytes, macrophages and endothelial cells) and they are used as a therapy in Multiple Sclerosis (MS).

**Figure 1:**
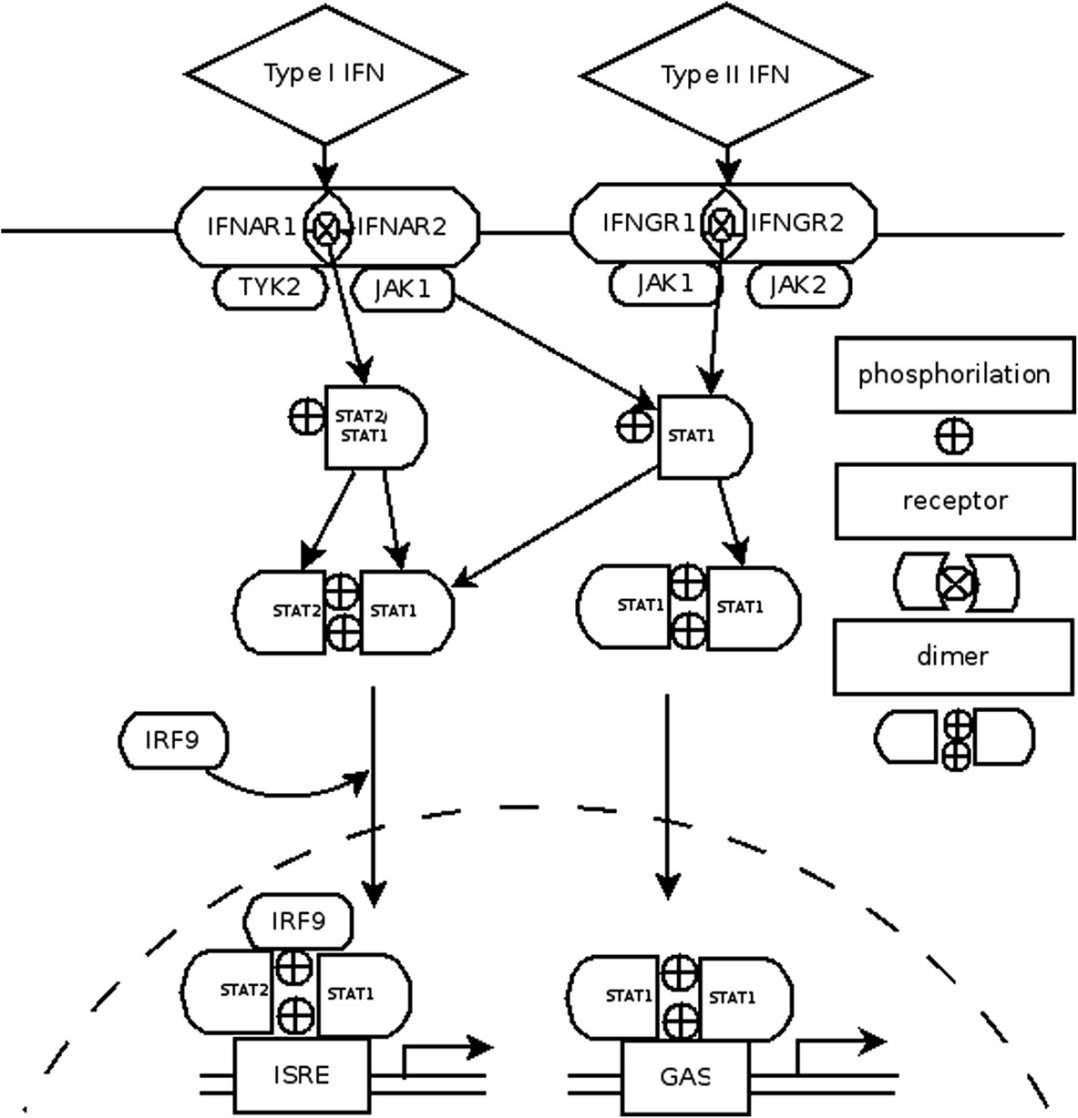
**The canonical type I and type II IFN signalling pathways.** The plot represents the canonical IFN pathways and the cross-talk between them, including the different pSTAT dimers formed after stimulation.

The canonical type I interferon (IFN) pathway involves different signaling cascades, one of which is the JAK/STAT pathway. This pathway is composed by several steps, which include receptor binding (IFNR1 and 2), transformation of the latent transcription factor (a protein of the STAT family) into its active form by phosphorylation, nuclear migration of the transcription factor (TF), binding of the TF to target promoters, and expression of their corresponding genes[1] (Figure 1). Previous studies have shown that phosphorylated STAT1 forms other TF complexes in response to type II interferons, the most important of which is a STAT1-STAT1 homodimer, known as GAF, that binds to IFN Gamma-activated sequence (GAS) elements [2].

The target genes of the IFN-beta pathway can be divided into three categories according to the type of activating transcription factor: 1) the ISGF3 complex activates genes containing an ISRE binding site in their promoter (e.g. ISG15, Mx1, OAS1, IRF7). 2) The GAF complex activates genes containing a GAS binding site in their promoter, such as SOCS1 and IRF1 [3,4]. 3) A third class of STAT protein complexes activates other canonical pathways that exhibit crosstalk with the JAK/STAT pathway (such as PI3K, NFkB, MAPK) [5]. Recently it was shown that different immune cell subtypes respond differently to IFN-beta induction through activation of these different types of genes [6].

Different proteins regulate STAT1 phosphorylation. Importantly, a negative feedback loop upon STAT1 activation coexists with a positive feedback mechanism. First, the phosphorylation of STAT1 is inhibited by its inhibitor SOCS1 [7]. The SOCS1 protein then inhibits STAT1 phosphorylation at the kinase level. Besides this negative loop based on SOCS1, STAT1 is a subject to positive regulation via the TF IRF1, whose transcription is induced by activated STAT1. IRF1 promotes the expression of the STAT1 gene at the transcriptional level. Given the existence of these multiple feedback loops, a mathematical modeling of the system would help provide an understanding of the response to type I IFN-beta.

Here we analyzed the type-I IFN-beta signaling pathway in macrophages, showing that the response of this pathway to IFN-beta stimulation takes the form of transient oscillations in STAT1 phosphorylation. We characterized and identified the critical elements governing the transient dynamics of IFN activation, and examined the influence of this dynamical regime in the response to IFN-beta.

Dynamical models of IFN induction of the JAK/STAT signaling pathway based on nonlinear ordinary differential equations, have been previously used to study the effect of IFN pre-treatment on the response of the immune system to virus infection [8,9] and the robustness of the pathway to noise and parameter fluctuations [10], among other problems. Systems biology approaches have also been applied to this pathway in order to examine its role in certain pathological mechanisms underlying the behavior of cancer cells [11], and its interaction with other key signaling pathways [8,12]. Here we combine our theoretical model with experimental observations. Our results show that a combination of positive and negative feedback loops, together with the eventual degradation of the IFN signal in the medium, leads to a transient oscillatory response in several components of the pathway. This behavior is consistent with previous numerical results found in pure modeling studies [13], and goes beyond previous observations that indicate a simpler transient response [10,14,15]. We interpret the transient oscillatory response of the pathway in terms of the potential effectiveness of IFN-beta treatment in MS patients.

## Results

### IFN-beta induces a transient oscillatory activation of the STAT1 pathway

It is well known that microbial and viral infections induce endogenous IFN-beta release by macrophages as part of the immune cell system response. We could observe IFN-beta production accompanied with significant increases in levels of phosphorylated STAT1 in the murine macrophage-like cell line RAW 264.7 stimulated with lipopolysaccharide (LPS) endotoxin and also, with viral fragments (poly(I:C)) (data not shown). In this study we focused on STAT1 signaling by IFN-beta stimulation in macrophages by challenging the RAW cell line with increasing concentrations of mouse IFN-beta.

**Figure 2:**
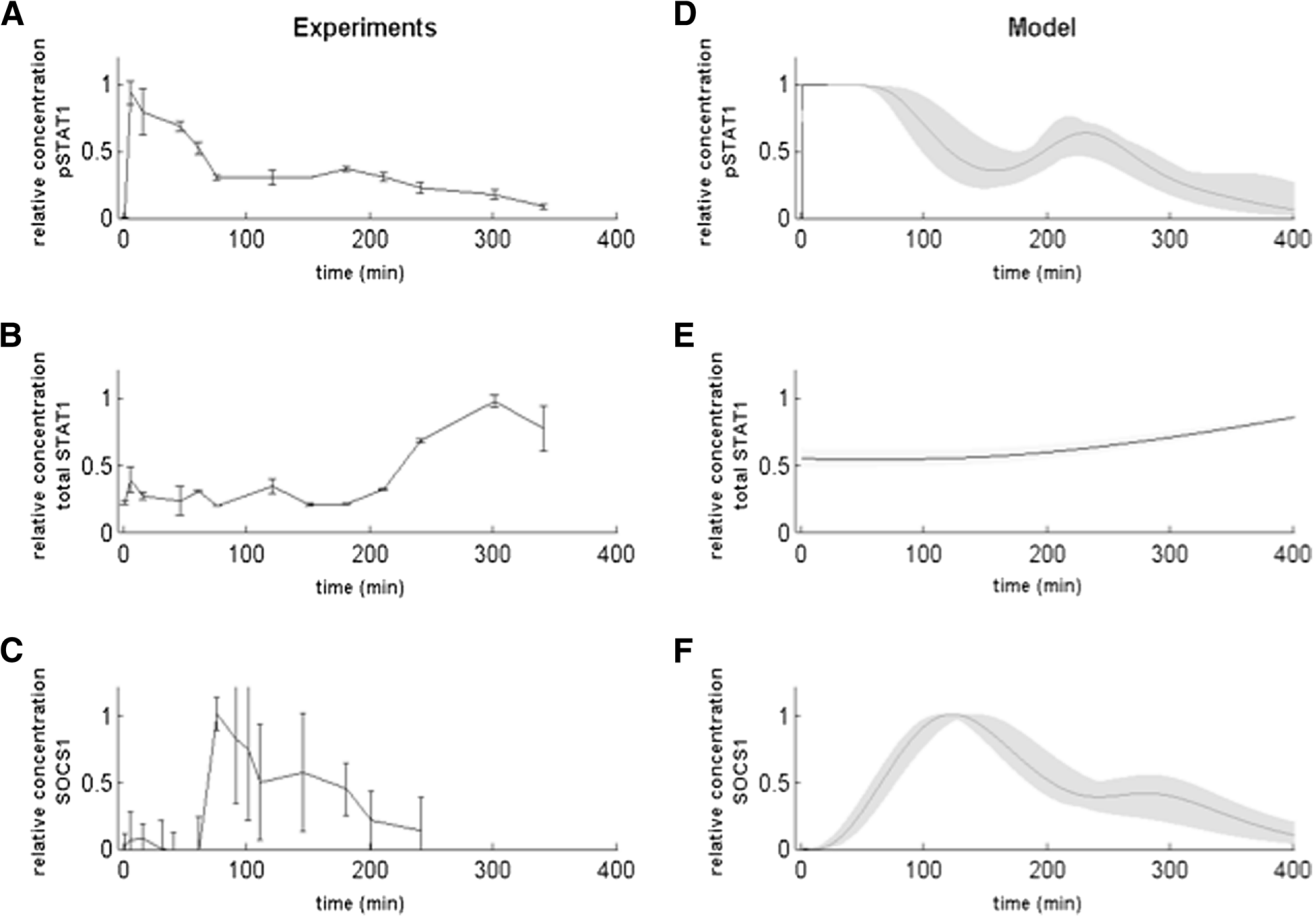
**Activation of type I IFN pathway by IFN-beta in Raw cell line and the corresponding simulations from the ODE model. A-C)** pSTAT1, STAT1 and SOCS1 protein concentration in cell lysates were measured by Luminex, Flow cytometry or ELISA after stimulation with IFN-beta (1000 units/ml). The data was normalized to the maximum level. **A)** pSTAT1 protein concentration in the Raw cells stimulated with IFN-beta; **(B)** Total STAT1 protein concentration in the Raw cells stimulated with IFN-beta; **C)** SOCS1 protein concentration in the Raw cells stimulated with IFN-beta. We plot here one out of three independent experiments, which were performed in duplicates. **D-F)** Model simulation and sensitivity analysis with ± 20% change for all parameters (shaded areas) **(D**: pSTAT1; **E**: STAT1; **F**: SOCS1**)**.

**Figure 3:**
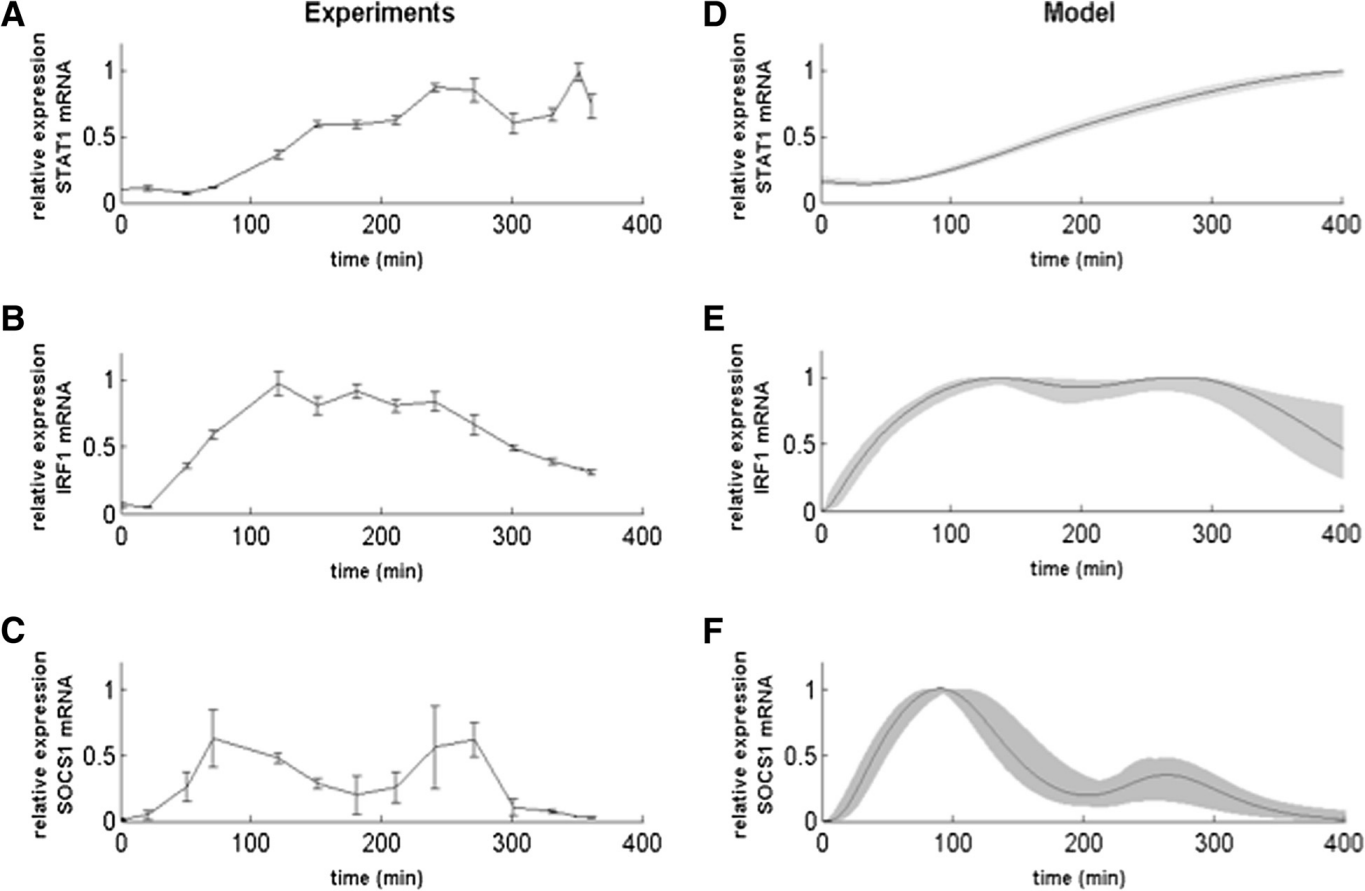
**Gene expression levels of STAT1, IRF1 and SOCS1 after IFN-beta stimulation and the corresponding simulations from the ODE model**. **A-C)** STAT1, IRF1 and SOCS1 RNA protein concentration in cell lysates were measured by RT-PCR in the Raw cells stimulated with IFN-beta (1000 units/ml); **A)** STAT1 RNA levels in the Raw cells stimulated with IFN-beta; **B)** IRF1 RNA levels in the Raw cells stimulated with IFN-beta; **C)** SOCS1 RNA levels in the Raw cells stimulated with IFN-beta. We plot here one out of three independent experiments, which were performed in duplicates. **D-F)** Model simulation and sensitivity analysis with ± 20% change for all parameters (shaded areas) for STAT1 **(D)**, IRF1 **(E)**, and SOCS1 **(F)** RNA levels.

We observed that phosphorylated STAT1 levels increased rapidly after IFN-beta induction (Figure 2A). The increase was significant as soon as 2 min after stimulation and reached a maximum at 10–15 min after stimulation, followed by a decrease that correlates with a substantial increase in the concentration of the SOCS1 protein (Figure 2C). A second, smaller peak was visible at around 180 min, followed by a subsequent decrease back to the baseline level after around 360 min. The quick decrease of phosphorylated STAT1 levels is in agreement with previous studies pointing to the activation of the negative feedback loop mediated by SOCS1 protein, which suppresses the phosphorylation of JAK1 and TYK2 proteins and prevents the formation of STAT-dimers [16]. The total level of STAT1 protein, on the other hand, is maintained practically constant until around 200 min after stimulation, after which it starts to increase slowly until the end of the experiments (Figure 2B). STAT1 mRNA levels grew quickly and continuously, starting sharply at around 75 min and leveling off after 200 min (Figure 3A). This increase in the mRNA level of STAT1, as soon as 1 hour after the induction of response by IFN-beta, agrees with the influence of the positive feedback loop IRF1 – STAT1 [17] and confirms the importance of this circuit for the pathway dynamics.

To analyze the expression of regulatory genes of the type I IFN pathway, we measured the levels of two downstream STAT1 genes, SOCS1 (responsible of the negative feedback) and IRF1 (mediator of the positive feedback). We observed an oscillatory behavior of SOCS1 mRNA during the first 360 minutes after stimulation, with clear peaks at around 90 min and 250 min, before returning to baseline levels (Figure 3C). On the other hand, IRF1 shows different dynamics, with its concentration raising quickly between 30 and 120 min, then reaching a plateau and decreasing more slowly after 250 min (Figure 3B). We also quantified the expression levels of other downstream effector IFN-induced genes, such as MX1 and OAS1b, but did not identify any activation of their transcription in the RAW 264.7 cell line after IFN-beta stimulation (data not shown). These observations are in agreement with a differential signal transduction mechanism in macrophages when compared to canonical JAK-STAT pathway in lymphocytes [4,18]. Our observations show that in the RAW 264.7 cell line the main activated genes were the ones controlled by the STAT1-STAT1 homodimer (IRF1 and SOCS1) and containing GAS elements in their promoter region. These genes are mainly responsible for the antimicrobial activity of the cells [19].

### Modeling the oscillatory signaling of type I IFN pathway

**Figure 4:**
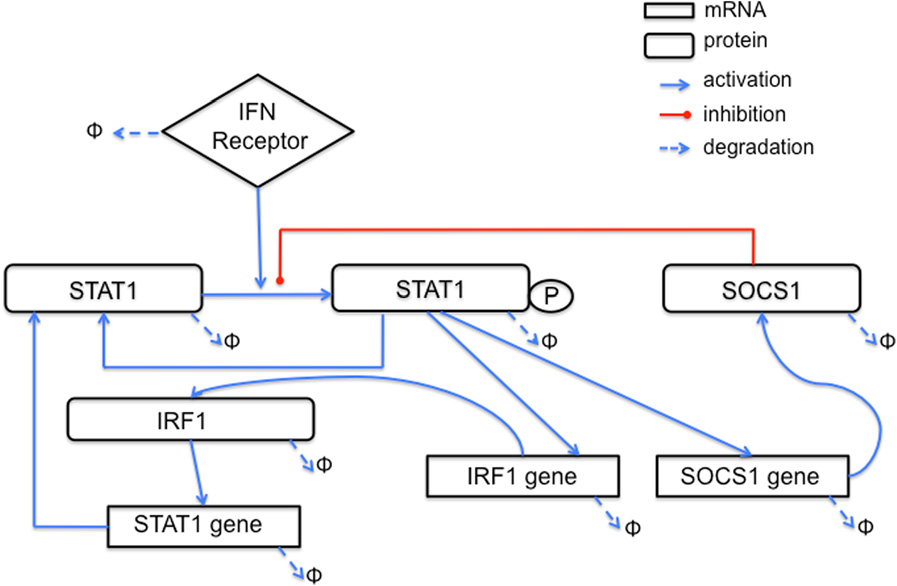
**Mathematical model of type I IFN signaling pathway.** Graphical representation of the biological processes modeled.

In order to identify the critical elements of the signaling pathway that are responsible for the transient oscillatory behavior observed experimentally in macrophages upon IFN-beta stimulation, we built an ODE model based in biological knowledge and experimental data (Figure 4). To minimize its complexity, we pursued the minimal system explaining the experimental observations, instead of a full descriptive system [14]. The model was not used to reproduce sustained endogenous IFN activation after viral infection, although it could be applied to that scenario.

The first ingredient of our model is the binding of IFN-beta to the receptor (with the concentration of activated receptor being represented by the variable *S* below). The activated receptor induces phosphorylation of the STAT1 protein (represented by *A*). Phosphorylated STAT1 translocates from the cytoplasm (*A*_*pc*_) to the nucleus (*A*_*pn*_) (Figure 4). In the nucleus, pSTAT1 complexes activate the transcription of SOCS1 and IRF1 genes. SOCS1 mRNA (*r*) is translated into SOCS1 protein (*R*), which inhibits further phosphorylation of STAT1. IRF1 mRNA (*f*) is translated to IRF1 protein (*F*), which activates the transcription of the STAT1 gene (with STAT1 mRNA being denoted by *a*). Each of the species has a certain linear degradation rate. We also introduced receptor internalization through an effective degradation (or deactivation) term, consistent with the literature [20]. With those ingredients, the model reads:

$$\mathrm{dS}/\mathrm{dt}=b_{s}-\lambda_{s}S$$

$$\begin{array}{c} \mathrm{dA}/\mathrm{dt}=b_{\exp}A_{\mathrm{pn}}+b_{\mathrm{deph}}A_{\mathrm{pc}}\\ \;+b_{A}a-\frac{b_{\mathrm{ph}}S\;A/k_{A}}{1+A/k_{A}+{\left(R/k_{I}\right)}^{q}}-\lambda_{\mathrm{STAT}}A \end{array}$$

$$\begin{array}{c} dA_{\mathrm{pc}}/\mathrm{dt}=\frac{b_{\mathrm{ph}}S\;A/k_{A}}{1+A/k_{A}+{\left(R/k_{I}\right)}^{q}}-b_{\mathrm{imp}}A_{\mathrm{pc}}-b_{\mathrm{deph}}A_{\mathrm{pc}}\\ \;-\lambda_{\mathrm{STAT}}A_{\mathrm{pc}} \end{array}$$

$$dA_{\mathrm{pn}}/\mathrm{dt}=b_{\mathrm{imp}}A_{\mathrm{pc}}-b_{\exp}A_{\mathrm{pn}}-\lambda_{\mathrm{STAT}}A_{\mathrm{pn}}$$

$$\mathrm{dr}/\mathrm{dt}=b_{r}\frac{{\left(A_{\mathrm{pn}}/k_{r}\right)}^{n}}{1+{\left(A_{\mathrm{pn}}/k_{r}\right)}^{n}}-\lambda_{r}r$$

$$\mathrm{dR}/\mathrm{dt}=b_{R}r-\lambda_{R}R$$

$$\mathrm{df}/\mathrm{dt}=b_{f}\frac{{\left(A_{\mathrm{pn}}/k_{f}\right)}^{m}}{1+{\left(A_{\mathrm{pn}}/k_{f}\right)}^{m}}-\lambda_{f}f$$

$$\mathrm{dF}/\mathrm{dt}=b_{F}f-\lambda_{F}F$$

$$\mathrm{da}/\mathrm{dt}=b_{a}\frac{{\left(F/k_{F}\right)}^{u}}{1+{\left(F/k_{F}\right)}^{u}}-\lambda_{a}a+B_{\mathrm{STAT}}$$

The parameters correspond to transcription rates, including basal transcription of STAT (*b*_*a*_*, b*_*r*_*, b*_*f*_*, B*_*STAT*_), translation rates (*b*_*A*_*, b*_*R*_*, b*_*F*_), cooperativity indexes (Hill coefficients, *n, m, q, u*), degradation rates (*λ*_*i*_), receptor activation (*b*_*S*_) and deactivation (*λ*_*S*_) rates, phosphorylation and dephosphorylation rates (*b*_*ph*_*, b*_*deph*_), and nucleo-cytoplasmic transport rates (*b*_*imp*_*, b*_*exp*_*)*. We adjusted some of the parameters using published data sources (Table 1), and the rest were estimated by manual fit of the model dynamics to the experimental data (Table 2) [21]. Initial conditions are listed in Table 3. The model implements the SOCS1-mediated negative feedback loop on pSTAT1 by means of a competitive inhibition term in the expression determining the phosphorylation rate of STAT1 in the equation for *A*_*pc*_, with the parameter *k*_*I*_ quantifying the half-maximal inhibition threshold and the exponent *q* defining the sharpness of the inhibition. Similarly, the positive feedback through IRF1 is described by the transcription activation term of STAT1 mRNA in the equation for *a*, with *k*_*F*_ representing the half-maximal activation threshold.

**Table 1:** Parameter values obtained from the literature

**Parameter**	**Value**	**Reference**
*b*_*deph*_	15 min half-life	[36,37]
*n*	3	[38]
*u*	1	[39]
*λ*_*r*_	2.82 hour half-life	This work
*λ*_*f*_	1.23 hour half-life	[40]
*A* initial	10^5^ molecules/cell	[41]
*S* initial	700-900 receptors/cell	[42]
*λ*_*STAT protein*_	24 hour half-life	[43]
*λ*_*F*_	30 min half-life	[44]
*f* initial	1 molecule/cell	[45]

**Table 2:** Parameters for type I IFN ODE model

**Name**	**Symbol**	**Value**	**Unity**
Translation rate for STAT1	*b*_*A*_	65	min^-1^
Receptor production rate	*b*_*S*_	0	min^-1^
Basal STAT1 RNA	*B*_*STAT1*_	0.0062	min^-1^
Transcription rate for STAT1	*b*_*a*_	0.1	min^-1^
Transcription rate for SOCS1	*b*_*r*_	12.8	min^-1^
Transcription rate for IRF1	*b*_*f*_	2.7	min^-1^
Translation rate for IRF1	*b*_*F*_	1.0 · 10^1^	min^-1^
Translation rate for SOCS1	*b*_*R*_	1.0 · 10^2^	min^-1^
Phosphorylation STAT1 rate	*b*_*ph*_	1.3 · 10^3^	min^-1^
Dephosphorylation STAT1 rate	*b*_*deph*_	0.036	min^-1^
Import to the nucleus rate (pSTAT1)	*b*_*imp*_	0.013	min^-1^
Export from the nucleus rate (STAT1)	*b*_*exp*_	0.048	min^-1^
STAT1 phosphorylation activation (Hill’s constant; half maximal activation)	*k*_*A*_	4680	molecules
Dissociation constant for the enzyme-inhibitor by SOCS1 (Hill’s constant; half maximal activation)	*k*_*I*_	82680	molecules
SOCS1 transcription activation by nuclear pSTAT1 (Hill’s constant; half maximal activation)	*k*_*r*_	23400	molecules
IRF1 transcriptional activation by pSTAT1	*k*_*f*_	7366	molecules
STAT1 transcriptional activation by IRF1	*k*_*F*_	1.3 · 10^5^	molecules
Cooperativity of SOCS1 protein over STAT1 dimers	*q*	4	
Cooperativity of STAT1 on SOCS1 gene promoter	*n*	3	
Cooperativity of STAT1 on IRF1 gene promoter	*m*	2	
Cooperativity of IRF1 on STAT1 gene promoter	*u*	1	
Receptor internalization/degradation rate	*λ*_*S*_	0.0229	min^-1^
SOCS1 RNA degradation rate	*λ*_*r*_	0.0347	min^-1^
SOCS1 protein degradation rate	*λ*_*R*_	0.0231	min-1
IRF1 RNA degradation rate	*λ*_*f*_	0.0173	min^-1^
IRF1 protein degradation rate	*λ*_*F*_	0.0116	min^-1^
STAT1 RNA degradation rate	*λ*_*a*_	0.0058	min^-1^
STAT1 protein degradation rate	*λ*_*STAT*_	0.0007	min^-1^

**Table 3:** Initial conditions for type I IFN model simulations

**Name**	**Symbol**	**Value**
Non-phosphorylated STAT1	*A*	1.0 · 10^5^ molecules
IFN activation receptor	*S*	1000 molecules
Phosphorylated nuclear STAT1	*Apn*	1 molecule
Phosphorylated cytoplasmic STAT1	*Apc*	10 molecules
STAT1 mRNA	*a*	1 molecule
IRF1 mRNA	*f*	1 molecule
SOCS1 mRNA	*r*	1 molecule
IRF1 protein	*F*	1 molecules
SOCS1 protein	*R*	1 molecule

The right panels in Figures 2 and 3 show simulation results corresponding to the experimental observations presented in the left panels. For comparative purposes, both the experimental and model variables were shown in relative concentrations dividing by their maximum value along the time series. We also performed a sensitivity analysis by simulating changes of ±20% for every model parameter, leading to deviations from the basal curve falling within the shaded areas shown in Figures 2 and 3.

The model simulations reproduce the main features observed experimentally, such as the first and very fast peak of phosphorylated STAT1 shortly after IFN-beta stimulation, and the second peak of smaller amplitude at around 200 min (Figure 2D). The concentration of total STAT1 protein is almost constant during the first 200 min, after which the protein level increases slowly (Figure 2E), following the increased expression level of STAT1 mRNA, which mRNA grows slowly starting at around 50 min after stimulation (Figure 3D). In turn, SOCS1 mRNA levels increase from the beginning, showing a first peak at around 90 min and a second smaller peak following the second peak of phosphorylated STAT1 at around 250 min (Figure 3F), in agreement with the experiments. Again similarly to the experiments, IRF1 mRNA levels show a bell-shaped time course (Figure 3E), with an increase resembling that of SOCS1 mRNA levels (Figure 3F) and remaining high from around 90 min to 250 min, when IRF1 mRNA levels decrease to half their maximum value at around 350 min.

**Figure 5:**
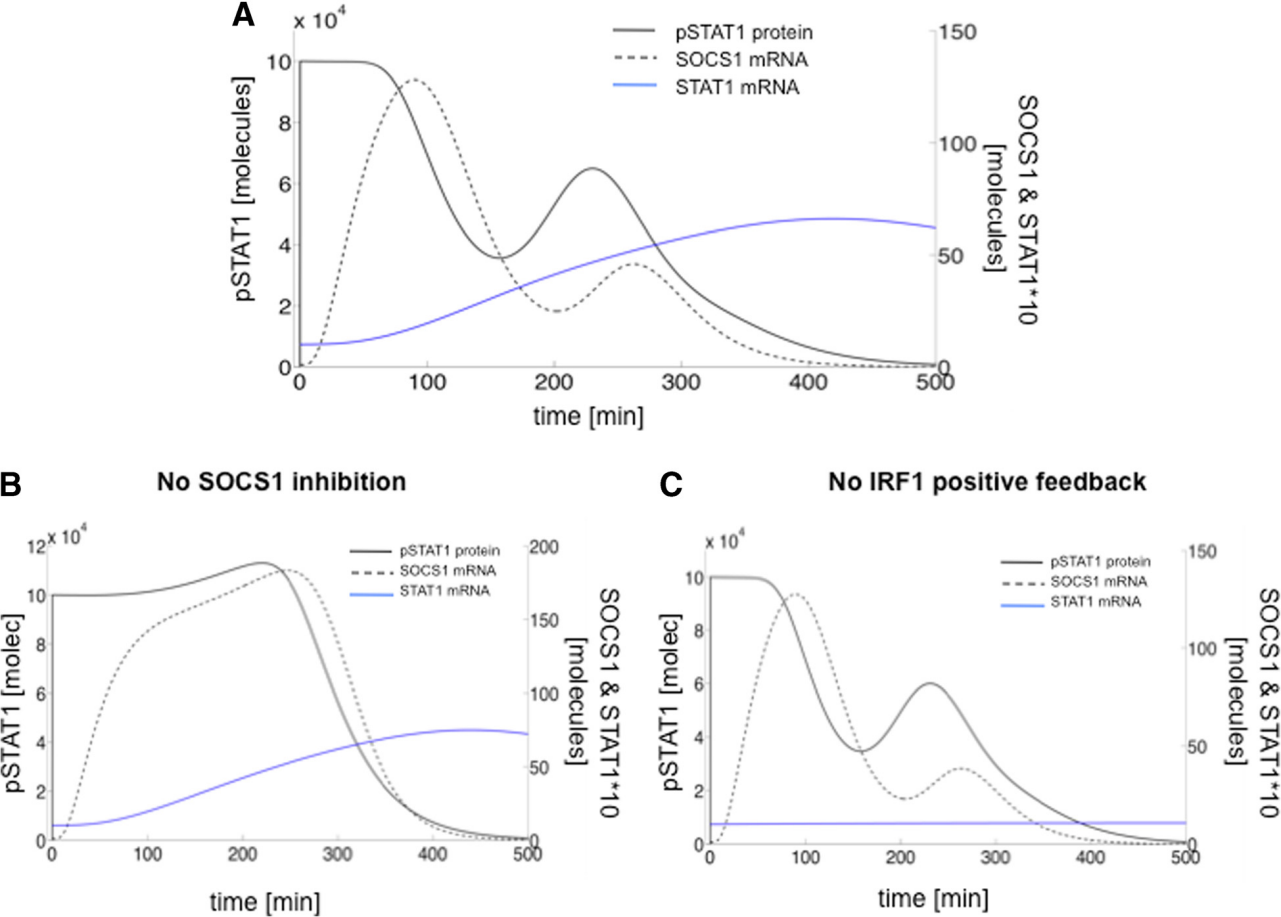
**Type I IFN pathway model simulations during the first 8 hours after IFN-beta stimulation. (A)** Model simulations showing oscillations of total (nuclear plus cytoplasmic) pSTAT1 protein, SOCS1 mRNA expression (dashed line) and STAT1 mRNA expression (dotted line). **(B)** Corresponding simulations where the SOCS1-mediated negative feedback is disrupted by assuming an infinite value of the repression threshold *k*_*I*_. **(C)** Corresponding simulations not including the IRF1-mediated positive feedback, by assuming a zero value of the STAT1 activation threshold *k*_*F*_ (which leads to saturation of the corresponding Hill function, so that the dependence of *a* expression on *F* is removed).

The model allows us to interpret the second peak observed experimentally in pSTAT1 and SOCS1-mRNA levels in terms of an underlying damped oscillatory dynamics. We now ask what are the mechanisms leading to oscillations, on the one hand, and to damping, on the other hand. A well-known gene circuit architecture that leads to oscillatory behavior is a combination of positive and negative feedback loops [22]. As mentioned above, our model contains a negative feedback loop mediated by SOCS1. We can examine in the model the effect of not having this feedback by eliminating SOCS1 signaling from the model. The results show that this negative feedback is required for the oscillatory behavior to arise: its absence leads to a transient plateau of high pSTAT1 levels during the first 4–5 h of IFN treatment (Figure 5B), which contrasts with the relaxation oscillator behavior obtained for our basal parameter values (Figure 5A), which is a closer match to the experimental observations (Figures 2–3). The model also contains a positive feedback loop mediated by IRF1. This loop, however, does not appear to be crucial for the oscillatory behavior of pSTAT1 (Figure 5C), and is only necessary to reproduce the experimentally observed increase of STAT1 expression (Figure 3A D).

**Figure 6:**
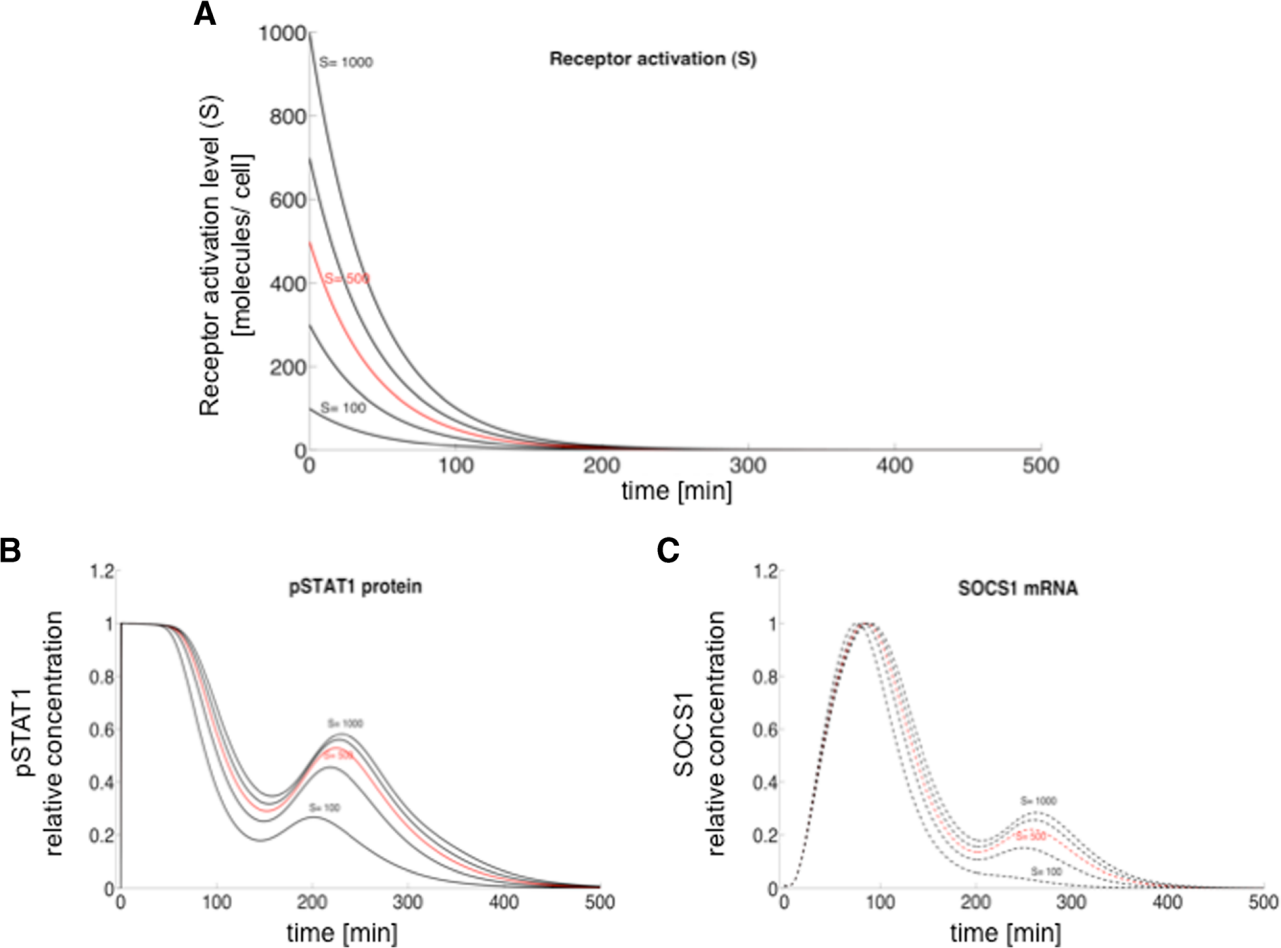
**Influence of activated receptor level on the transient oscillatory dynamics. (A)** Time evolution of the activation level of type I IFN receptors in the model (*S*) by stimulation with IFN-beta (added at *t = 0*) for different initial levels (in red, *S = 500* molecules at *t = 0*). **(B, C)** pSTAT1 **(B)** and SOCS1 mRNA **(C)** dynamic responses for varying levels of initial receptor activation as in panel **A** (lower lines in **B** and **C** correspond to lower lines in **A**).

The combination of negative and positive feedbacks discussed in the preceding paragraph would naturally lead to sustained oscillatory behavior. The experimental observations shown in Figures 2 and 3, however, reveal a strong damping of the oscillations that lead to their sudden disappearance. This behavior is not consistent with the standard damping undergone by nonlinear oscillations when they become unstable via a Hopf bifurcation, in which case the damping is either slow close to the bifurcation, or the damped oscillations are too weak to begin with far away from the bifurcation. A key distinctive characteristic of our model is the fact that the external input to which the system is subject (mediated by the activated receptors represented by *S* in the model above) decays monotonously due to receptor inactivation by internalization or degradation [20,23]. Assuming a linear decay, the external input decreases exponentially (Figure 6A), taking the system quickly out of the oscillatory regime and thus leading to a strong damping of the oscillations, as seen experimentally. Exploring systematically the dependence of the dynamics on the receptor level, we observed that as the initial levels of activated receptor decrease (Figure 6A) the second peak of both pSTAT1 (Figure 6B) and SOCS1 mRNA (Figure 6C) levels diminish, with the SOCS1 expression peak disappearing earlier than the pSTAT1 concentration peak.

### Bifurcation analysis of the STAT1 pathway model identifies translocation to the nucleus as a critical step

The temporal evolution of the phosphorylation of STAT1 can be crucial for understanding the response to IFN-beta therapy, and may provide an explanation of the lack of response to this therapy in some cases [24]. In particular, transient oscillatory dynamics could provide a way for the STAT1 pathway to increase the duration of its response to IFN-beta in a physiological manner (i.e. without a period of sustained constant activation as in Figure 5B). In order to establish the conditions under which this transient dynamics exists, we analyzed the behavior of the system for combinations of two-parameter pairs, distinguishing between the parameter values for which pSTAT dynamics is overdamped (and thus non-oscillatory) and those for which the oscillations are underdamped (which corresponds to the experimental situation reported above). We focused on the phosphorylation and dephosphorylation rates (*b*_*ph*_ and *b*_*deph*_) and export and import rates (*b*_*exp*_ and *b*_*imp*_). These parameters represent crucial steps to regulate the nuclear availability of transcription factors such as pSTAT dimers, and thus also the expression of downstream genes.

**Figure 7:**
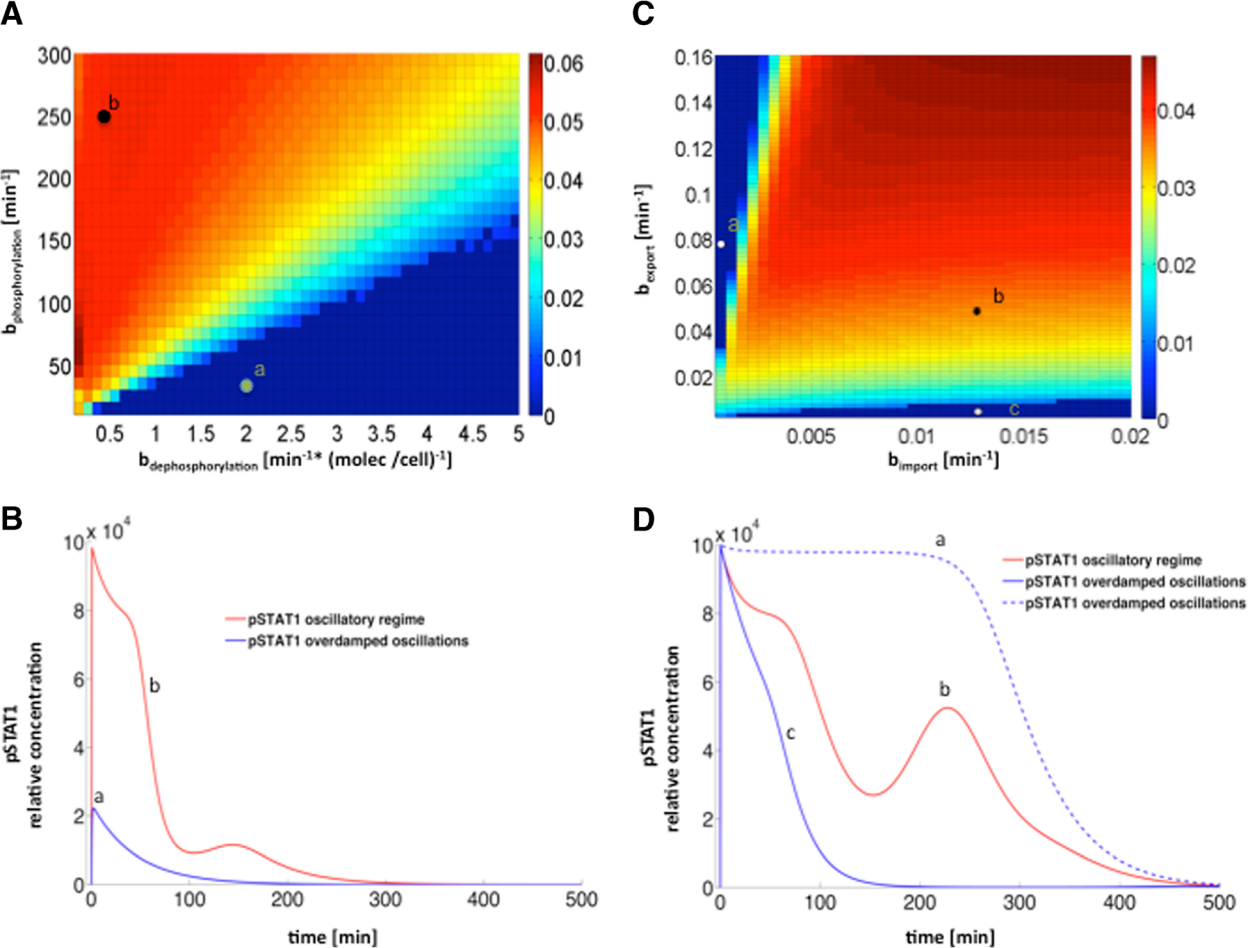
**Stability analysis of the steady state solution in two 2D parameter spaces. (A, C)** The color scale represents the absolute value of the imaginary part of the stability eigenvalue with maximum real part, corresponding to the steady state of the system after IFN stimulation, for varying phospho/ dephosphorylation rates (*b*_*ph*_ and *b*_*deph*_, panel **A)**, and nuclear export/import rates (*b*_*exp*_ and *b*_*imp*_, panel **B)**. Two distinct dynamic regimes can be identified with this analysis: the damped oscillatory regime (shifted to red) and the overdamped/ non-oscillatory regime (blue). **(B, D)** Examples of pSTAT1 time evolution in both regimes (damped and over-damped in red/blue lines, respectively) corresponding to parameter position of circle markers for the two diagrams shown in panels **A** and **B**, respectively.

In order to identify mathematically the two different dynamical regimes mentioned above, we examined the stability properties of the steady state of the ODE system for a constant activated receptor level (*S*) equal to its initial value. In that context, the underdamped/oscillatory regime is characterized by a steady state that takes the form of an unstable focus (i.e. the stability eigenvalue with maximum real part has negative imaginary part), whereas the steady state in the overdamped/non-oscillatory regime is a node (the stability eigenvalue with maximum real part has no imaginary component). In that way, by calculating the imaginary part of the stability eigenvalue of the steady state with maximum real part, we can identify the parameter regions in which the pathway exhibits a transient oscillatory response to IFN-beta. The result, for the parameter space formed by the phosphorylation and dephosphorylation rates *b*_*ph*_ and *b*_*deph*_, is shown in Figure 7A. This figure shows, on the one hand, the prevalence of oscillations for a wide range of these parameters, and on the other hand it tells us the conditions for which transient oscillations exist. For instance, increasing sufficiently the dephosphorylation rate can transform an oscillatory regime into a non-oscillatory one, and reversely, by making the phosphorylation rate large enough the system can be made to exhibit transient oscillations. Figure 7B shows examples of these two dynamical regimes for two specific parameter sets within this phase diagram.

We also tested the influence of the import rate of pSTAT1 molecules into the nucleus, and of the export rate of STAT1 from the nucleus into the cytosol. By tuning both parameters (*b*_*exp*_ and *b*_*imp*_) simultaneously, we observed again that the transient oscillatory regime is prevalent in this system (Figure 7C). We found that the oscillatory regime is associated with high nuclear import rates in combination with high export rates. For high export rates but low import rates, the pathway exhibits an overdamped (non-oscillatory) response, showing a sustained plateau in the transient level of pSTAT1 (discontinuous blue line in Figure 7D). Conversely, for high import rates and low export rates the response is also overdamped, but with a faster decay (continuous blue line in Figure 7D).

## Discussion

The aim of this work was to characterize the dynamics of the key components of the type I IFN-beta signaling pathway in macrophage RAW 264.7 cells. This system robustly translates extracellular chemical signals through cell membrane receptors, leading to phosphorylation of the STAT transcription factors, which induce gene expression of multiple targets. JAK/STAT signaling directly regulates the immune system response under viral or bacterial infection, and is also important in autoimmune diseases and cancer treatments. The IFN signaling network affects different complex pathways, involving processes such as differentiation, proliferation, survival and cell death. Importantly, it is a canonical pathway involved in first-line treatments of multiple sclerosis as a main target of the IFN system [25] but, also, affects different complex pathways, involving processes such as differentiation, proliferation or survival and cell death [26,27].

In this paper, we used a combination of experimental approaches in order to obtain a quantitative picture of the response of the JAK/STAT signaling pathway to IFN-beta stimulation, and to identify the most relevant aspects of its dynamics to be modeled with kinetic equations. Experiments uncovered several important features of JAK/STAT signaling dynamics during the first eight hours after treatment with IFN-beta. For example, our results showed the transient oscillatory nature of STAT1 activation (pSTAT1), with a fast increase in cytosol concentration early after stimulation (within the first hour), followed by a secondary concentration peak at around 200 min. A key STAT1 transcription target such as SOCS1 also showed two peaks of expression (correlated in time to the pSTAT peaks) at around 90 min and 250 min after stimulation, whereas another important target, namely IRF1, exhibited a more bell-shaped plateau signal, respectively (Figures 2 and 3). Our model simulations also exhibit a transient oscillatory behavior in pSTAT1 concentration, and reveal that the oscillations require the presence of a negative feedback loop on STAT1, mediated by its phosphorylation inhibitor SOCS1. Previous mathematical models of the type I and type II IFN pathways have suggested the possibility that STAT1 pathway has an oscillatory behavior [9,13] and indicated the importance of the SOCS1 negative feedback [10,14,28,29]. Another factor that has been proposed to be important in defining the response to IFN is the basal level of receptors of the JAK/STAT pathway [30]. In our model this aspect was also taken into account, showing clear effects on the dynamics of the pathway response (Figure 6). Going beyond previous models, our theoretical results show that the physiological regime of the pathway’s response to IFN-beta takes the form of damped oscillations that can be identified by means of a stability analysis of the model’s steady state solution. This analysis shows that processes such as the phosphorylation and dephosphorylation of pSTAT1, and the transport of STAT1 between the nuclear and cytosol compartments, can make the pathway switch between underdamped and overdamped oscillatory regimes [31].

### Implications of the type I IFN signaling dynamics in autoimmune diseases

IFN-beta is the most common treatment for MS [32], exerting a pleiotropic immunomodulatory activity not well understood [25]. IFN-beta treatment decreases activation, proliferation, cytokine release, and migratory properties of activated T cells, diminishing their ability to enter and damage the brain tissue. In spite of these properties, up to 40% of patients do not respond to IFN-beta therapy, which represents a significant health problem [24]. Previous genomic studies have identified certain genes belonging to the IFN pathway that are associated with a lack of response to IFN-beta, suggesting that the genetic background of certain individuals may modulate this pathway, and consequently the response to therapy, by specific transcriptional profiles [33]. For example, it was recently shown that the response to IFN-beta differs between immune cells, and an analysis of non-responders to IFN-beta therapy indicates an impairment of the type I IFN pathway in the monocytes of those patients [6,34].

Our study indicates the importance of identifying the temporal dynamics of the concentration of certain key components of the JAK-STAT pathway, such as the phosphorylated form of the STAT1 protein, and of the expression of interferon-stimulated transcription genes like SOCS1 and IRF1, within the first 8 hours of IFN-beta administration. Cataloguing these dynamics could provide us with early molecular biomarkers that allow us to distinguish the lack of response to IFN-beta therapy of certain MS patients.

## Methods

### Materials and reagents

Cells were obtained from ATCC library, mouse recombinant IFN-beta was purchased from Cell sciences, lipopolysaccharide from Escherichia coli and poly(I:C) salt was purchased from Sigma-Albrich, lipofectamine 2000, Hiperfect transfection agent were purchased from QIAGEN, Taqman PCR master mix, VIC-dye GAPDH endogenous control, IRF1, SOCS1, STAT1, STAT2, MX1, OAS1a pre-designed FAM-dye assays were purchased from Applied Biosystems, total STAT1 and STAT1(pTyr701) antibodies and beads, cell detection kit for xMAp assays were purchased from Merck Millipore (Billerica). Alexa Flour 647 STAT1 (pTyr701) and PE STAT1 N-terminal anti-Mouse antibodies and all buffers for cytometry were purchased from BD biosciences. APC-labelled IFNAR1 antibody was purchased from Biolegend.

### Cell culture and stimulation

Mouse leukemic monocyte macrophage cell line RAW 264.7 cell line was purchased from ATCC and maintained in DMEM medium complemented with 10% fetal bovine serum and 1% antibiotics at 37°C and 5% CO2. The cells were passed every 2–3 days and maintained in 20-80% surface coverage. One day before the stimulation the cells were seeded in 12 well plates in concentration 1 × 10^6^ cells/well. The cells were stimulated with 1000 units of recombinant mouse IFN-beta, 15 μg of LPS for different times or 15 μg of poly (I:C) solution. At the end of stimulation supernatants or cells were collected for further analysis. The same amount of PBS was added at the corresponding time-points to the control samples.

### RT-PCR

Cell lysates were prepared with QiaShredder columns and total RNA was isolated using standard Qiagen Rnaesy Mini kit protocol. Equal amount of total RNA was added to each reverse transcription reaction tube (High-Capacity cDNA Reverse Transcription Kit from Applied Biosystems and cDNA was used for a second step of RT-PCR. Results were analyzed using relative 2CTT method normalized to a GAPDH endogenous control (VIC-dye primer-limited control from Applied Biosystems) as described before [35]. All the qRT-PCR experiments were performed in triplicates and repeated three times independently.

### Western blot and quantification

Western blot (WB) was performed using polyclonal rabbit anti-mouse pSTAT1 and STAT1 N-terminal antibodies (Abcam) using standard WB protocol. Western blot results were quantified using ImageJ software (http://rsb.info.nih.gov/ij/index.html) using the method of Luke Miller (http://www.lukemiller.org/journal/2007/08/quantifying-western-blots-without.html)

### ELISA and xMAP multiplexing assays

IFN-beta in culture supernatants and SOCS1 protein concentration in cell lysates were measured by standard sandwich ELISA with anti-mouse SOCS1 antibodies (Abcam). IRF1 protein concentration in cell lysates was measured by in-cell ELISA using the kit (Thermo Scientific) STAT1 total protein and phosphorylated state (Tyr701) concentrations (nuclear and cytoplasmic together) were measured using xMAP assays and read in Luminex 201 platform using standard vacuum separation protocol (Millipore). xMAP experiments were repeated twice.

#### *Flow cytometry*

Cells for flow cytometry were stimulated with 1,000 μn/ml of IFN-beta as stated before and fixed immediately after stimulation. IFNAR1 receptor on the surface of the RAW 264.7 cells was marked using APC-labelled anti-IFNAR antibody. The mean fluorescent intensity was calculated using FlowJo software. For STAT1 staining cells were fixed immediately after stimulation in order to preserve phosphorylation and then permeabilized using Perm III buffer (BD biosciences). Samples were stained simultaneously with anti-STAT1 (pTyr701) and anti-STAT1 total (N-terminus) antibodies. The mean fluorescent intensity, the percent of staining-positive cells, the medians and the standard deviation were calculated using FlowJo software and the raw single-cell data were extracted to plot the histograms and further analysis.

#### *Mathematical modeling*

The model and simulations were run in MATLAB using the ODE15s solver (Matlab codes are provided in the Additional files 1, 2 and 3). The stability analysis of the dynamical system was performed with custom-made Matlab codes.

### Availability of supporting data

The model is available as a matlab script in the supporting materials. The raw experimental data are available from the authors upon request.

## Abbreviations

ODE: ordinary differential equations; STAT1: Signal Transducers and Activators of Transcription 1; SOCS1: Suppressor of cytokine signaling 1; IRF1: Interferon regulatory factor 1; AAF: IFNA-activated-factor; ISRE: Interferon-sensitive response element; GAS: Interferon-Gamma Activated Sequence; ISGF3: IFN-stimulated regulatory factor 3; IFNbeta: interferon beta; IFNg: interferon gamma; LPS: Lipopolysaccharide; poly(I:C): Polyinosinic:polycytidylic acid; RT-PCR: quantitative reverse transcription polymerase chain reaction; GAPDH: Glyceraldehyde 3-phosphate dehydrogenase; FAM: Fluorescein amidite; ELISA: Enzyme-Linked Immunosorbent Assay; OAS1a - 2'-5': oligoadenylate synthetase 1 gene; MX1: Interferon-induced GTP-binding protein gene.

## Competing interests

The authors declare no competing interests.

## Authors’ contributions

Study design: IP, PV, JGO, NDP; In vitro experiments: IP; Mathematical model: NDP, EA, IP; Analysis of the data: EA, IP, NDP; Writing article: PV, IP, EA, JGO. All authors read and approved the final manuscript.

## Supplementary Material

Additional file 1 Type I IFN pathway ODE model equations. Click here for file

Additional file 2 Type I IFN pathway ODE model as Matlab file. Click here for file

Additional file 3 Integration and solutions of the Type I IFN pathway ODE model as Matlab file. Click here for file
