# Supplementary material for: Transient oscillatory dynamics of interferon beta signaling in macrophages
Source: BMC Syst Biol. 2013 Jul 9;7:59. doi: 10.1186/1752-0509-7-59 (PMC3711797; doi:10.1186/1752-0509-7-59)
Supplement: Additional file 1 — Type I IFN pathway ODE model equations. [file 1752-0509-7-59-S1.docx]

% ODE model

function dy = model_IFN(t,y,p)

% variable names:

S = y(1);

A = y(2);

Ap_c = y(3);

Ap_n = y(4);

r = y(5);

R = y(6);

f = y(7);

F = y(8);

a = y(9);

% parameter names:

b_S = p(1);

b_exp = p(2);

b_imp = p(3);

b_ph = p(4);

b_deph = p(5);

k_A = p(6);

k_I = p(7);

k_r = p(8);

k_f = p(9);

k_F = p(10);

b_A = p(11);

b_r = p(12);

b_R = p(13);

b_f = p(14);

b_F = p(15);

b_a = p(16);

lambda_S = p(17);

lambda_r = p(18);

lambda_R = p(19);

lambda_f = p(20);

lambda_F = p(21);

lambda_a = p(22);

q = p(23);

n = p(24);

m = p(25);

u = p(26);

lambda_stat = p(27);

basal_stat = p(28);

% equations:

dy = zeros(9,1);

% dy(1)=dS/dt: S active receptors (protein)

dy(1) = b_S - lambda_S*S;

% dy(2)=dA/dt: STAT1 non-phosphorylated form (protein)

dy(2) = + b_exp*Ap_n ...

+ b_deph*Ap_c ...

+ b_A*a ...

- b_ph*S*(A/k_A)/(1+(A/k_A)+power(R/k_I,q)) ...

- lambda_stat*A;

% dy(3)=dApc/dt: STAT1 phosphorylated form (protein)

dy(3) = + b_ph*S*(A/k_A)/(1+(A/k_A)+power(R/k_I,q)) ...

- b_imp* Ap_c ...

- b_deph* Ap_c...

- lambda_stat*Ap_c;

% dy(4)=dApn/dt: STAT1 phosphorylated form in cell nucleus (protein)

dy(4) = b_imp * Ap_c ...

- b_exp* Ap_n...

- lambda_stat*Ap_n;

% dy(5)=dr/dt: SOCS1 expression (mRNA)

dy(5) = + b_r * power(Ap_n/k_r,n)/(1+power(Ap_n/k_r,n)) ...

- lambda_r * r;

% dy(6)=dR/dt: SOCS1 (protein)

dy(6) = + b_R * r ...

- lambda_R * R;

% dy(7)=df/dt: IRF1 expression (mRNA)

dy(7) = + b_f * power(Ap_n/k_f,m)/(1+power(Ap_n/k_f,m)) ...

- lambda_f * f;

% dy(8)=dF/dt: IRF1 (protein)

dy(8) = + b_F * f ...

- lambda_F * F;

% dy(9)=da/dt: STAT1 expression (mRNA)

dy(9) = + b_a * power(F/k_F,u)/(1+power(F/k_F,u))-lambda_a * a+ basal_stat;

end

% Solution for ODE IFN_model (Integration)

% parameter values:

p= [0, ... % b_S

0.08,0.013,... % b_exp, b_imp

1300,0.036,... % b_ph, b_deph

4680,82680,... % k_A, k_I,

23400,7.3e+03,130e+03,... % k_r, k_f, K_F

65,... % b_A

12.8, 1.0e+02,... % b_r, b_R

2.7,10,... % b_f, b_F

1e-01,... % b_a

0.02,... % lambda_S

0.03, 0.02,... % lambda_r, lambda_R

0.017, 0.01,... % lambda_f, lambda_F

0.006,... % lambda_a

4, 3, 2, 1,... % q, n, m, u

6.9e-04, 0.006 % lambda_stat, basal_stat

];

tmax = 500;

lcolor='k';

options = odeset('MaxStep',1); % integration step limit

% Solving ODE model in matlab (using a solver for stiff type problems):

sol = ode15s(@model_IFN,[0 tmax],[1000 1e+05 10 1 1 1 1 1 1],options,p);

% Some graphs:

figure(); hold on;

plot(sol.x,(sol.y(3,:)),sprintf('%s-','b'),'LineWidth',1); hold on;

legend('pSTAT1 oscillartory regime');

ylabel('STAT1_p');

figure(); hold on;

subplot(6,1,1);

plot(sol.x, sol.y(1,:),sprintf('%s-',lcolor),'LineWidth',1); hold on;

legend('S');

ylabel('receptor');

subplot(6,1,2);

plot(sol.x,(sol.y(4,:)),sprintf('%s-','k'),'LineWidth',1); hold on;

legend('Ap_c+Ap_n');

ylabel('STAT1_p');

subplot(6,1,3);

plot(sol.x,(sol.y(2,:)+sol.y(3,:)+sol.y(4,:)),sprintf('%s-','k'),'LineWidth',1); hold on;

legend('A+Apc+Apn');

ylabel('total STAT1');

subplot(6,1,4);

plot(sol.x, sol.y(5,:),sprintf('%s-','k'),'LineWidth',1);hold on;

plot(sol.x, sol.y(7,:),sprintf('%s--','k'),'LineWidth',1);

ylabel('mRNA');

legend('r','f');

subplot(6,1,5);

plot(sol.x, sol.y(9,:),sprintf('%s:','k'),'LineWidth',1); hold on;

ylabel('mRNA');

legend('a');

subplot(6,1,6);

plot(sol.x, sol.y(6,:),sprintf('%s-',lcolor),'LineWidth',1); hold on;

plot(sol.x, sol.y(8,:),sprintf('%s--',lcolor),'LineWidth',1);

ylabel('downstream proteins');

legend('R','F');

xlabel('time (min)');

%the end
